# Enteric Fever in Campaigning

**Published:** 1901-07-27

**Authors:** 


### Enteric Fever in Campaigning.

Dr. Leigh Canney, a graduate in honours of the London University, and well known as a distinguished physician in Egypt, has lately put forth a pamphlet on the prevention of enteric fever in armies, to which a non-medical evening contemporary has given very prominent notice in its columns, and which is certainly calculated to appeal strongly, if not to the special knowledge of epidemiologists, at least to that class of lay reader whom it is customary to describe as "intelligent." The basis of Dr. Canney's proposal is that it should be made a point at once of duty as a matter of military discipline, and of honour as a matter bearing upon the credit and welfare of the regiment or other organisation of which every soldier forms part, that no unboiled water should be drunk in the course of a campaign; and he suggests that a sufficient number of men should be told off to form a "Water Corps," to attend every division or detachment of an army upon its march, or when it is going into action, and to supply the beverage in sufficient quantities to meet all reasonable or probable demands. The army is to lean upon the "Water Corps" for its drink; and nothing is to be drunk except what the Corps has served out. Dr. Canney enters into elaborate calculations with regard to the number of men who would be withdrawn from the fighting ranks, with regard to the weight of the water-purifying equipment, to consist mainly of boilers and petroleum, with cloths for such rough filtration as would be sufficient to remove suspended mud, and with regard to the number of mules or the amount of waggon transport that the arrangement would require; and he draws a contrast between these requirements and those incidental to the prevalence of enteric fever in a manner which leaves a large balance in favour of the former.

The late Reverend Robert Montgomery, who was once a popular preacher in London, and whose name is chiefly remembered from its having been preserved, like a fly in amber, in one of Macaulay's critical essays, is said to have entertained an invincible objection to monosyllables. Nevertheless he sometimes found it difficult to escape from them; and, on one occasion, he introduced the word "if" into a thrilling discourse. Pausing for a moment, he interjected an additional sentence, couched in the following words: "But here I should explain, to my less-instructed brethren, that 'if' is an inferential particle." Now in the case of Dr. Canney's proposals, there seems to us to be a very considerable "inferential particle" involved; and lest the proposals themselves should be eagerly seized upon by some of *les gobe-mouches* of the House of Commons, and made the basis of questions by such authorities as Mr. John Morley or Mr. MacNeill, we hasten to point out its nature. We do not in the least doubt the possibility of doing all that Dr. Canney suggests in the way of supplying boiled water; nor do we doubt that such a course would greatly diminish other demands upon transport, *if* only it were effective in preventing the occurrence of enteric fever. That is where the inferential particle comes in. The weak place of the argument is in the assumption that polluted water, although well known to be a common channel for the introduction of the enteric bacillus into the human body, can ever be regarded as the sole channel. "If" it were the sole channel, and if Dr. Canney's proposals were carried into effect, his name might live in history as that of one of the greatest benefactors, if not of the human race, at least of that portion of it which takes the field in armies. Unfortunately the evidence does not support any such assumption. We know that the bacillus will thrive and multiply in certain soils and in the dejections of the sick, and that such matters are liable to be sun-dried and widely dispersed in the form of dust or through the agency of insects. In the particular case of South Africa we know that fever prevailed there long before the war; that is, that it was a well-known disease of the country. In the great towns of England, in many or most of which the water supply of the population is now guarded against pollution with sedulous care, the effect has been to bring about a great diminution of enteric fever, obviously by the closure of one of its former channels of diffusion; but cases of the disease still occur, if not as frequently, yet quite as severely as before. It gets abroad in some other way. If the War Office were to follow Dr. Canney's advice, and to attach a Water Corps to every army or expeditionary force, it may fairly be assumed that water-borne enteric would disappear. What may also with some probability be assumed, in the present state of knowledge, is that the beneficial action of the Water Corps would be to a considerable extent frustrated by the unchecked diffusion of dried forms of the bacillus.

